# P66 Initiating antimicrobial stewardship rounds in an elective orthopaedic hospital—scoping and implementation: a joint MDT effort in orthopaedics

**DOI:** 10.1093/jacamr/dlaf230.073

**Published:** 2025-12-04

**Authors:** Nickisha Patel, Neil Jenkins

**Affiliations:** The Royal Orthopaedic Hospital NHS Foundation Trust, Birmingham, UK; The Royal Orthopaedic Hospital NHS Foundation Trust, Birmingham, UK

## Abstract

**Background:**

Antimicrobial stewardship (AMS) in elective surgical settings remains an underexplored domain, particularly in orthopaedics, a specialty with consistently high antimicrobial usage due to the nature of infections. In contrast to acute or high-risk environments, AMS opportunities in elective orthopaedic hospitals are often overlooked due to perceived low complexity and the existence of protocol-driven prophylaxis. However, despite this it still experiences the stewardship challenges of a secondary care facility. This project aimed to address this gap by introducing structured, multidisciplinary AMS ward rounds at a tertiary elective orthopaedic centre to evaluate current antimicrobial use, identify improvement opportunities and assess feasibility for sustainable integration.

**Objectives:**

To map existing antimicrobial prescribing practices in an elective orthopaedic inpatient setting; to categorize prescribing patterns and identify targets for intervention; to assess the feasibility, scope and clinical value of weekly structured AMS ward rounds; and to promote multidisciplinary collaboration and guideline adherence.

**Methods:**

Over a 6 month period (March–August 2025), weekly AMS rounds were conducted across five level-one wards and one high-dependency unit. Patients receiving antimicrobials were prospectively screened using the hospital's electronic prescribing system (PICS) and categorized into four predefined groups: Bone Infection Service—under existing specialist MDT care; excluded from AMS review; AMS—cases with nosocomial/community infections or off-guideline prescribing; Prophylaxis—perioperative antimicrobial use per established protocols; and Other—long-term antimicrobial prophylaxis (e.g. post-splenectomy). Cases in the AMS group were further triaged by the screening pharmacist, with appropriate interventions made or escalated to the AMS round. The AMS team comprised an Infectious Diseases Consultant, Antimicrobial Pharmacist and a Bone Infection Service nurse.

**Results:**

Across 14 AMS rounds, out of the 955 inpatients, 40% (*n*=382) were receiving antimicrobials at time of screening. Of these: 30% (*n*=114) were under Bone Infection Service care and excluded from intervention analysis; 38% (*n*=144) were receiving surgical prophylaxis, largely guideline compliant; and 22% (*n*=84) met criteria for AMS review due to complex, non-prophylactic antimicrobial use. Within the AMS cohort, 53 patients were discussed during rounds, resulting in 43 interventions, including de-escalation, initiation of appropriate therapy, or referral to MDT. Key themes included inconsistent documentation (e.g. vague postoperative antibiotic plans), off-guideline prescribing and treatment of clinically insignificant microbiology findings.

**Conclusions:**

This scoping initiative demonstrates that structured AMS rounds are both feasible and impactful in an elective orthopaedic setting. Despite being a low-acuity environment, 40% of inpatients were on antimicrobials, with 13% of patients on antimicrobials warranting targeted stewardship interventions. Pharmacist-led screening, coupled with multidisciplinary review, enabled efficient use of time and resources while fostering improved prescribing culture. The model identified practice variation, gaps in guideline coverage and areas for systemic improvement, laying the foundation for broader AMS integration. Future work will include outcome tracking, expansion of prophylaxis guidance and development of targeted education and guideline refinement strategies. A future focus on the Watch and Reserve categories, unravelling prophylaxis regimes, creating tailored education and training packages such as prescribing for sepsis will be aimed to be rolled out amongst staff.

